# Supplementary material for: Diels–Alder Adducts of Morphinan-6,8-Dienes and Their Transformations
Source: Molecules. 2022 Apr 30;27(9):2863. doi: 10.3390/molecules27092863 (PMC9102320; doi:10.3390/molecules27092863)
Supplement: Supplementary file 1 [file molecules-27-02863-s001.zip › molecules-1662015-supplementary.pdf]

Supplementary Material

Diels-Alder Adducts of Morphinan-6,8-dienes and their Transformations

János Marton [1]*, Anikó Fekete [2], Paul Cumming [3,4] Sándor Hosztafi [5], Pál Mikecz [2],

and

Gjermund Henriksen [6,7,8]*

[1] ABX advanced biochemical compounds Biomedizinische Forschungsreagenzien GmbH, Heinrich-Glaeser-Strasse 10-14, D-01454 Radeberg, Germany; marton@abx.de
[2] Division of Nuclear Medicine and Translational Imaging, Department of Medical Imaging, Faculty of Medicine, University of Debrecen, Nagyerdei krt. 98, H-4032 Debrecen, Hungary; fekete.aniko@science.unideb.hu (A.F.); mikecz.pal@unideb.hu (P.M.)
[3] Department of Nuclear Medicine, Bern University Hospital, Freiburgstraße 18, CH-3010 Bern, Switzerland; paul.cumming@insel.ch
[4] School of Psychology and Counselling, Queensland University of Technology, Brisbane, Australia
[5] Institute of Pharmaceutical Chemistry, Semmelweis Medical University, Hőgyes Endre utca 9, H-1092 Budapest, Hungary; hosztafi.sandor@pharma.semmelweis-univ.hu
[6] Norwegian Medical Cyclotron Centre Ltd., Sognsvannsveien 20, N-0372 Oslo, Norway; gjermund.henriksen@syklotronsenteret.no
[7] Institute of Basic Medical Sciences, University of Oslo, P. O. Box 1105, Blindern, N-0317 Oslo, Norway
[8] Institute of Physics, University of Oslo, Sem Saelands vei 24, N-0371 Oslo, Norway

ORCID ID

Dr. János Marton	0000-0001-9411-7899
Dr. Anikó Fekete	0000-0002-0951-6092
Prof. Dr. Paul Cumming	0000-0002-0257-9621
Dr. Sándor Hosztafi PhD, DSc	0000-0003-3793-4651
Dr. Pál Mikecz	0000-0003-4367-7460
Prof. Dr. Gjermund Henriksen	0000-0001-9608-9895

*** CORRESPONDENCE**

Dr. János Marton

ABX advanced biochemical compounds Biomedizinische Forschungsreagenzien GmbH
Heinrich-Glaeser-Strasse 10-14
D-01454 Radeberg, Germany
Email: marton@abx.de

Prof. Dr. Gjermund Henriksen

Norwegian Medical Cyclotron Centre Ltd.
Sognsvannsveien 20,
N-0372 Oslo, Norway
Email: gjermund.henriksen@syklotronsenteret.no
Phone: +47-950-351-78

CONTENTS

			PAGE
**1.**	**ABBREVIATIONS**		3
**2.**	**SUPPLEMENTARY FIGURES**		7
**2.1.**	**Figure S1**	Synthesis of ^{17}*N*-northevinone	7
**2.2.**	**Figure S2**	Reaction of 20-phenethyl-thevinol with diethyl azodicarboxylate	7
**2.3.**	**Figure S3**	*N*^{17}-demethylation of thevinone using the *von* Braun method	8
**2.4.**	**Figure S4**	The Hudlický route for preparation of buprenorphine methylether	8
**2.5.**	**Figure S5**	Synthesis of BPN methylether from 20-*tert*-butyl-dihydrothevinol using the *N*-demethylation/*N*-acylation reaction sequence	8
**2.6.**	**Figure S6**	Synthesis of buprenorphine from oripavine	9
**2.7.**	**Figure S7**	Synthesis of *N*^{17}-cyclopropylmethyl-nororipavine from oripavine	9
**2.8.**	**Figure S8**	Chemical structures of non-acid-sensitive 6,14-ethenomorphinans 3-*O*-demethylated with boron tribromide	9

1. Abbreviations

Compound, Term, Acronym	Comp.	Name, Synonyms
**6,14-amidoHap**	**251**	6,14-amido heroin hapten
**14-amidoMorHap**	**255**	14-amido morphine hapten
**14-amidoHerHap**	**256**	14-amido heroin hapten
**Banyu compound-24 (C-24)**	**-**	(2*R*)-1-(phenylmethyl)-*N*-(3-spiro[1*H*-2-benzofuran-3,4'-piperidine]-1'-ylpropyl) pyrrolidine-2-carboxamide; CAS RN: [475150-69-7]
**BBB**	**-**	blood brain barrier
**benzyne**	**-**	aryne; higly reactive species derived from an aromatic ring by removing two substituents
**BHT**	**-**	2,6-di-*tert*-butyl-4-methylphenol, 2,6-di-*tert*-butyl-*p*-cresol, CAS RN: [128-37-0]
**Boc**	**-**	*tert*-butoxycarbonyl group
**BPN**	**96**	buprenorphine, CAS RN: [52485-79-7]
**[^{11}C]BPN**	**460b**	[^{11}C]buprenorphine, [6-*O*-methyl-^{11}C]buprenorphine
**BrCN**	**-**	cyanogen bromide, CAS RN: [506-68-3]
**BU08028**	**101**	(5*R*,6*R*,7*R*,9*R*,13*S*,14*S*,20*S*)-17-cyclopropylmethyl-α-(1,1-dimethylpropyl)-4,5α-epoxy-18,19-dihydro-3-hydroxy-6-methoxy-α-methyl-6,14-ethenomorphinan-7-methanol, CAS RN: [1333904-22-5]
**BU127**	**396g**	*N*^{17}-cyclopropyl-20*S*-phenyl-dihydronororvinol; (5*R*,6*R*,7*R*,9*R*,13*S*,14*S*,20*S*)-17-(cyclopropylmethyl)-4,5α-epoxy-18,19-dihydro-3-hydroxy-6-methoxy-α-methyl-α-phenyl-6,14-ethenomorphinan-7-methanol; CAS RN: [1417783-62-0]
**BU128**	-	(5*R*,6*R*,7*R*,9*R*,13*S*,14*R*,20*R*)-17-(cyclopropylmethyl)-4,5-epoxy-3-hydroxy-6-methoxy-7-methyl-α-phenyl-6,14-ethenomorphinan-7-methanol CAS RN: [1417783-82-4]
**BU10112**	**-**	(1′*R*,5α,6R,7R,14α)-1′-(3-methylphenyl-1′-(4,5α-epoxy-7,8-dihydro-3-hydroxy-6-methoxy-7β-methyl-17-cyclopropylmethyl-6,14-ethenomorphinan-7-yl)-methan-1′-ol, CAS RN: [1417783-97-1]
**BU10119**	**102**	(5*R*,6*R*,7*R*,9*R*,13*S*,14*S*,20*R*)-17-(cyclopropylmethyl)-4,5-epoxy-18,19-dihydro-3-hydroxy-6-methoxy-7-methyl-α-phenyl-6,14-ethenomorphinan-7-methanol CAS RN: [1417783-94-8]
**BU10120**	**103**	(5*R*,6*R*,7*R*,9*R*,13*S*,14*S*,20*R*)-17-(cyclopropylmethyl)-4,5-epoxy-3-hydroxy-6-methoxy-7-methyl-α-(4-fluorophenyl)-6,14-ethenomorphinan-7-methanol CAS RN: [1417783-90-4]
**Caf**		carfentanil, 1-(2-phenylethyl)-4-[(1-oxopropyl)phenylamino]-4-piperidine carboxylic acid methyl ester, R-31,833, 4-carboxymethyl-fentanyl, CAS RN: [59708-52-09]
**[^{11}C]Caf**		[^{11}C]carfentanil, CAS: [122402-12-4]
**Cbz**	**-**	benzyloxycarbonyl group, Z
**CNS**	**-**	central nervous system
**COSY**	**-**	correlated spectroscopy, two-dimensional shift correlations via spin-spin coupling
**CPCO**	**-**	cyclopropylcarbonyl group
**CPM**	**-**	cyclopropylmethyl group
**Cypre**	**-**	cyprenorphine, RX285M, 17-cyclopropylmethyl-4,5-epoxy-3-hydroxy-6-methoxy-α,α-dimethyl-6,14-ethenomorphinan-7-methanol, CAS RN: [44065-22-8]
**DA reaction**	**-**	Diels-Alder reaction
**DAMGO**	**-**	Tyr-*D*-Ala-Gly-(*N*Me)Phe-Gly-ol
**DBU**	**-**	1,8-diazabicyclo[5.4.0]undec-7-ene, CAS RN: [6674-22-2]
**DBN**	**-**	1,5-diazabicyclo[4.3.0]non-5-ene, CAS RN: [3001-72-7]
**DEAD**	**-**	diethyl azodicarboxylate, CAS RN: [1972-28-7]
**DHE**		dihydroetorphine, CAS RN: [14357-76-7]
**DIBAL**	**-**	diisobutylaluminum hydride CAS RN: [1191-15-7]
**dihydrothevinone**	**104**	4,5α-epoxy-18,19-dihydro-17-methyl-3,6-dimethoxy-7α-acetyl-6,14-ethenomorphinan, CAS RN: [16196-82-0]

Compound, Term, Acronym	Comp.	Name, Synonyms
**DIPEA**	-	ethyldiisopropylamine, *N,N*-diisopropylamine, Hünig's base
**9,10-DMA**	201	9,10-dimethylantracene, CAS RN: [781-43-1]
**DMAD**	-	dimethyl acetylenedicarboxylate, CAS RN: [762-42-5]
**DMAP**	-	*N,N*-dimethylpyridin-4-amine, 4-dimethylaminopyridine, CAS RN: [1122-58-3]
**DMF**	-	*N,N*-dimethylformamide
**DMP**	-	Des-Martin periodane
**DMSO**	-	dimethyl sulfoxide, $(CH_3)_2SO$, CAS RN: [67-68-5]
**δ-OR**	-	δ-opioid receptor
**DPDPE**	-	[*D*-Pen^2,*D*-Pen^5]enkephalin, CAS RN: [88373-73-3]
**DPN**	95	diprenorphine, Revivon, M5050, CAS RN: [14357-78-9]
**[^{11}C]DPN**	460a	[^{11}C]diprenorphine, [6-*O*-methyl-^{11}C]diprenorphine
**ED_{50}**	-	effective dose
**EDG substituent**	-	electron donating substituent
**EDTA**	-	ethylenediaminetetracetic acid, CAS RN: [60-00-4]
**ε-OR**	-	ε-opioid receptor
**EP**	-	ethyl propiolate, ethyl acetylenecarboxylate, CAS RN: [623-47-2]
**EWG substituent**	-	electron withdrawing substituent
**FAO**	300	fumaramidooripavine
**[^{18}F]FcyF**	-	[^{18}F]cyclofoxy, 6-deoxy-6*β*-[^{18}F]fluoro-naltrexone, CAS RN: [103223-58-1]
**FE-BPN**		6-*O*-(2-fluoroethyl)-6-*O*-desmethyl-buprenorphine
**FE-DPN**		6-*O*-(2-fluoroethyl)-6-*O*-desmethyl-diprenor phine, CAS RN: [1391522-66-9]
**FE-PEO**		6-*O*-(2-fluoroethyl)-6-*O*-desmethyl-phenethyl-orvinol
**[^{18}F]FE-BPN**	471	6-*O*-(2-[^{18}F]fluoroethyl)-6-*O*-desmethyl-buprenorphine
**[^{18}F]FE-DPN**	468	6-*O*-(2-[^{18}F]fluoroethyl)-6-*O*-desmethyl-diprenorphine
**[^{18}F]FP-*nor*BPN**	467	*N*^{17}-(3-[^{18}F]fluoropropyl)-*nor*-buprenorphine
**[^{18}F]FP-*nor*DPN**	466b	*N*^{17}-(3-[^{18}F]fluoropropyl)-*nor*-diprenorphine
**[^{18}F]FE-PEO**	470	6-*O*-(2-[^{18}F]fluoroethyl)-6-*O*-desmethyl-phenethyl-orvinol
**[^{18}F]FEOTos**	-	2-[^{18}F]fluoroethyl tosylate
**β -FNA**	-	β -funaltrexamine
**GPI**	-	Guinea-pig ileum
**GR103545**	-	[(3,4-dichlorophenyl)acetyl]-(3*R*)-(1-pyrrolidinylmethyl)-1-piperazine carboxylic acid methyl ester
**[^{11}C]GR103545**	-	[(3,4-dichlorophenyl)acetyl]-(3*R*)-(1-pyrrolidinyl methyl)-1-piperazine carboxylic acid methyl-^{11}C ester
**HATU**	-	1-[bis(dimethylamino)methylene]-1*H*-1,2,3-triazolo[4,5-b]pyridinium-3-oxide hexafluorophosphate, CAS RN: [148893-10-1]
**HDA reaction**		hetero Diels-Alder reaction
**HMPA**		hexamethylphosphoramide, CAS RN: [680-31-9]
**HOMO**		highest occupied molecular orbital
**IA-DPN**		iodoallyl-diprenorphine
**9-I-9BBN**	-	9-iodo-9-borabicyclo[3.3.1]nonane, CAS RN: [70145-42-5]
**IDA**	-	iminodiacetic acid, CAS RN: [142-73-4]
***iso*-BPN**	-	3-*O*-methyl-6-*O*-desmethyl-buprenorphine
**JDTic**	-	1,2,3,4-tetrahydro-7-hydroxy-*N*-[(1*S*)-1-[[(3*R*,4*R*)-4-(3-hydroxyphenyl)-3,4-dimethyl-1-piperidinyl]methyl]-2-methylpropyl]-(3*R*)-isoquinolinecarboxamide, CAS RN: [361444-66-8]
**κ-OR**	-	κ-opioid receptor
**K_i**	-	inhibition constant
**KHMDS**	-	potassium bis(trimethylsilyl)amide, CAS RN: [40949-94-8]
**LDA**	-	lithium diisopropylamide, $LiN[CH(CH_3)_2]$, CAS RN: [4111-54-0]
**LH**	-	Luteinizing hormone
**L-Selectride**	-	Lithium-tri-*sec*-butylborohydride
**LTA**	-	lead tetraacetate, CAS RN: [546-67-8]
**LUMO**	-	lowest unoccupied molecular orbital

Compound, Term, Acronym	Comp.	Name, Synonyms
**M99**	**97**	etorphine, Immobilon, CAS RN: [14521-96-1]
**M320**	**-**	isopentyl orvinol
***m*CPBA**	**-**	3-chloroperbenzoic acid, CAS RN: [937-14-4]
**16α-Me-Cypre**	**-**	16α-methylcyprenorphine, 17-cyclopropylmethyl-4,5α-epoxy-3-hydroxy-6-methoxy-α,α,16α-trimethyl-6,14-etheno morphinan-7-methanol, CAS RN: [40994-80-7]
**[^{11}C]MeNTI**	**-**	*N*1′-[^{11}C]methyl-naltrindole
**μ-OR**	**-**	μ-opioid receptor
**MORV**	**-**	20-methylorvinol
**MP**	**-**	methyl propiolate, methyl acetylenecarboxylate, CAS RN: [922-67-8]
**MTBD**	**-**	1-Methyl-2,3,4,6,7,8-hexahydro-1*H*-pyrimido[1,2-a]pyrimidine CAS RN: [84030-20-6]
**MVD**	**-**	mouse vas deferens
**Naloxone**	**-**	N^{17}-allyl-14-hydroxy-dihydromorphinone, N^{17}-Allyl-noroxymorphone, CAS RN: [465-65-6]
**Naltrexone**	**-**	N^{17}-cyclopropylmethyl-14-hydroxy-dihydromorphinone, N^{17}-cyclopropylmethyl-noroxymorphone, NTX, CAS RN: [16590-41-3]
**NBS**	**-**	*N*-bromosuccinimide
**NCS**	**-**	*N*-chlorosuccinimide
**NCA**	**-**	no-carrier-added
**nepenthone**	**16b**	7α-benzoyl-4,5α-epoxy-17-methyl-3,6-dimethoxy-6,14-ethenomorphinan CAS RN: [464-66-4]
**nociceptine**	**-**	Phe-Gly-Gly-Phe-Thr-Gly-Ala-Arg-Lys-Ser-Ala-Arg-Lys-Leu-Ala-Asn-Gln
**NOP**	**-**	nociceptin/orphanin receptor
***nor*BNI**	**-**	norbinaltorphimine, CAS:[105618-26-6]
**NTI**	**-**	naltrexone-indole, naltrindole, CAS RN: [111555-53-4]
**ORs**	**-**	opioid receptors
**PEO**		20*R*-phenethyl-orvinol, CAS RN: [14521-98-3]
**[^{11}C]PEO**		[6-*O*-methyl-^{11}C]phenethyl-orvinol
**PET**	**-**	positron emission tomography
**PTAD**	**-**	4-phenyl-4*H*-1,2,4-triazoline-3,5-dione
**RIA**	**-**	radioimmunoassay
**RVD**	**-**	rat vas deferens
**SLL-004C**	**422c**	*p*-amino-nepenthone, CAS RN: [2084834-43-3]
**SLL-020ACP**	**390a**	N^{17}-cyclopropylmethyl-nornepenthone, CAS RN: [188340-60-5]
**SLL-039**	**434**	*N*-cyclopropylmethyl-7α-[4′-(*N*′-benzoyl)amino-phenyl]-6,14-endoethano-tetrahydronorthebaine
**SLL-1206**	**437j**	*N*-cyclopropylmethyl-7α-[3′-(p-methoxybenzyl)amino-phenyl]-6,14-endoethano-tetrahydronorthebaine
**SLL-603**	**430**	N^{17}-cyclopropymethyl-7α-methyl-dihydronornepenthone CAS RN: [2246405-55-8]]
**SPECT**	**-**	single-photon emission computed tomography
**TAN-821**	**333**	17-(cyclopropylmethyl)-4,5-α-epoxy-3,6-dihydroxy-*N*-methyl-*N*-(2--phenylethyl)-6,14-ethenomorphinan-7-α-carboxamide, CAS RN: [214064-71-8]
**TAN-1014**	**335**	(4b*S*,8*R*,8a*R*,9a*S*,13a*R*,13b*R*)-7-(cyclopropylmethyl)-5,6,7,8,9,9a,11,12-octahydro-1-hydroxy-11-(2-phenylethyl)-10*H*,13b*H*,8a,13a-etheno-4,8-methanobenzofuro [3,2-h]pyrido[3,4-g][1,3]benzoxazine-10-one, CAS RN: [757232-46-5]
**TBDMS**	**-**	*tert*-butyldimethylsilyl group
**TBTU**	**-**	O-(Benzotriazol-1-yl)-*N*,*N*,*N*',*N*'-tetramethyluronium tetrafluoroborate CAS RN: [125700-67-6]
**TDBPN**	**461b**	3-*O*-trityl-6-*O*-desmethyl-buprenorphine, CAS RN: [157891-93-5]
**TDDPN**	**461a**	3-*O*-trityl-6-*O*-desmethyl-diprenorphine, CAS RN: [157891-92-4], «*Luthra-precursor*», TDDPN was the first product of the company *ABX advanced biochemical compounds* Biomedizinische Forschungsreagenzien GmbH, Radeberg in 1997

Compound, Term, Acronym	Comp.	Name, Synonyms
**TE-TDPEO**	**469**	6-*O*-(2-tosyloxyethyl)-6-*O*-desmethyl-3-*O*-trityl-phenethyl-orvinol, CAS RN: [1614258-10-4]
**TDPEO**	**463**	3-*O*-trityl-6-*O*-desmethyl-phenethyl-orvionol, CAS RN: [1187551-69-4]
**thevinone**	**16a**	4,5*α*-epoxy-17-methyl-3,6-dimethoxy-7*α*-acetyl-6,14-ethenomorphinan, CAS RN: [15358-22-2]
**thienorphine**	**100**	(5*R*,6*R*,7*R*,9*R*,13*S*,14*S*,20*R*)-17-cyclopropylmethyl-4,5*α*-epoxy-18,19-dihydro-3-hydroxy-6-methoxy-*α*-methyl-*α*-[2-(2-thienyl)ethyl]-6,14-ethenomorphinan-7-methanol, CAS RN: [852100-94-8]
**TMS**	**-**	trimethylsily group
**TMEDA**	**-**	tetramethylethylenediamine, CAS RN: [110-18-9]
**Tos**	**-**	tosyl group
**Tr**	**-**	trityl group; triphenylmethyl group
**Troc**	**-**	2,2,2-trichloroethoxycarbonyl group
**TT**	**-**	tetanus oxide
**U69,593**	**-**	(+)-*N*-methyl-2-phenyl-*N*-[(5*R*,7*S*,8*S*)-7-pyrrolidin-1-yl-1-oxaspiro[4.5]decan-8-yl]acetamide, CAS RN: [96744-75-1]

2. Supplementary figures (to section 2.3.6.)

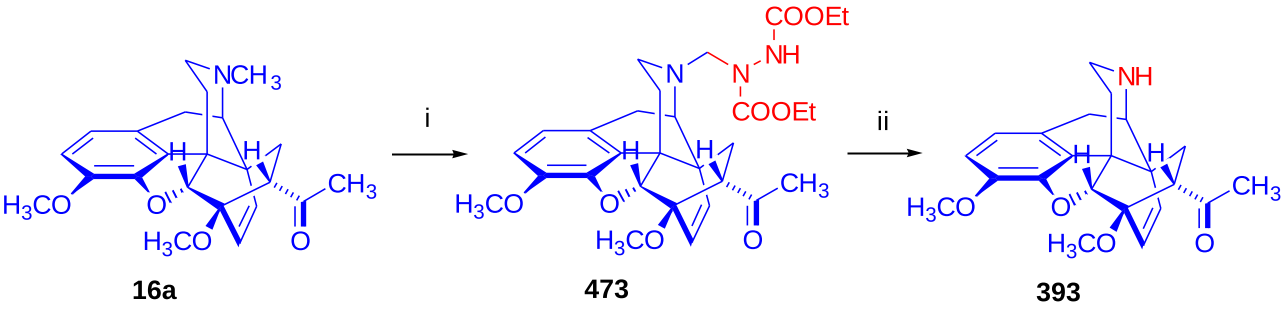

2.1. Figure S1 Synthesis of [17]*N*-northevinone

Reagents and conditions: (i): diethyl azodicarboxylate, benzene, reflux, 7 h; (ii): pyridinium chloride, EtOH, room temperature, 8 h

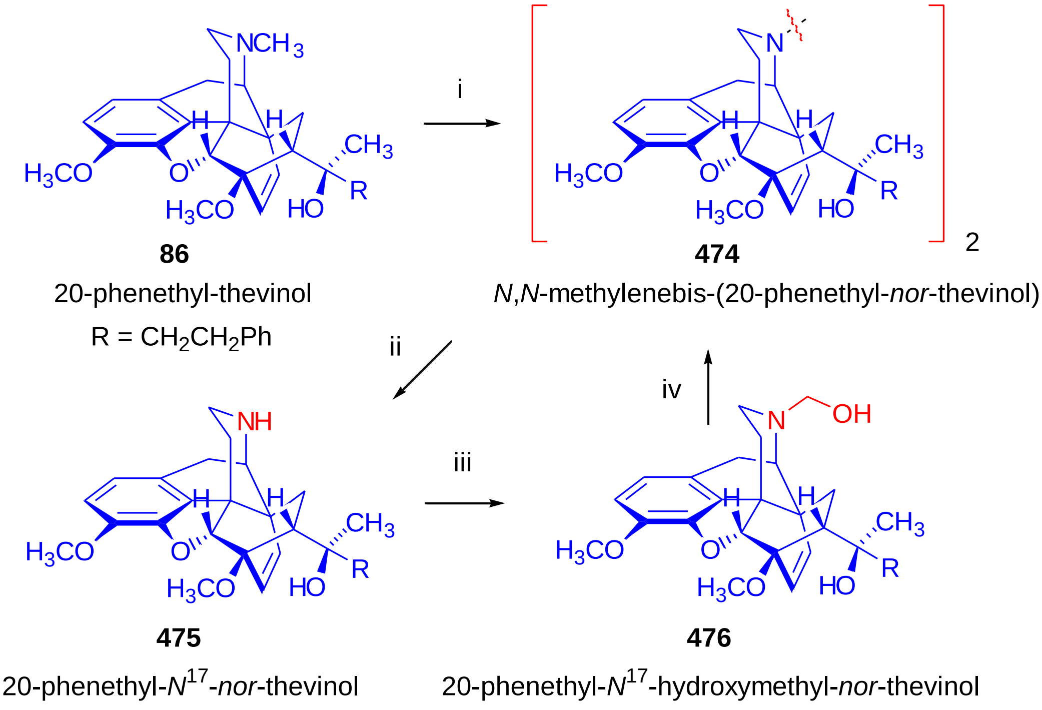

2.2. Figure S2 Reaction of 20-phenethyl-thevinol with diethyl azodicarboxylate

Reagents and conditions: (i): diethyl azodicarboxylate, acetone, reflux 1h; (ii): 6% acetic acid, Δ; (iii): 30% aqueous formaldehyde, EtOH, 45 °C, 30 min; (iv): **A**. EtOH, Δ, or **B**. **476**, EtOH, Δ

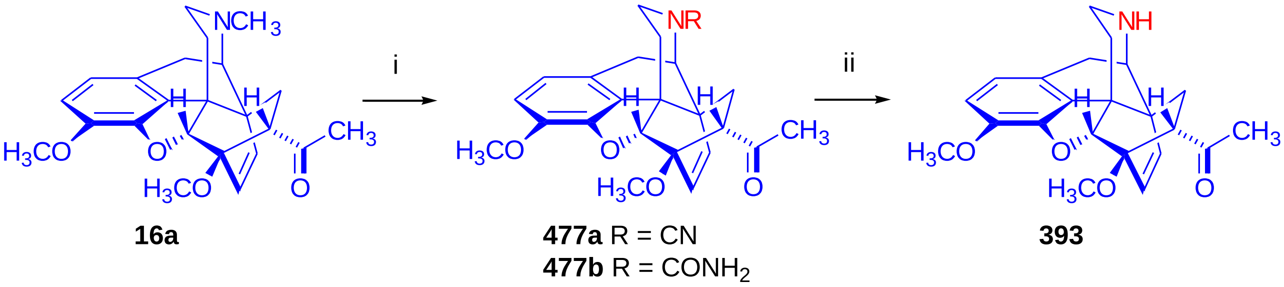

2.3. Figure S3 *N*[17]-demethylation of thevinone using the *von* Braun method

Reagents and conditions: (i): BrCN, $CHCl_3$, reflux, 12 h; (ii): 2 M HCl, reflux, 2 h

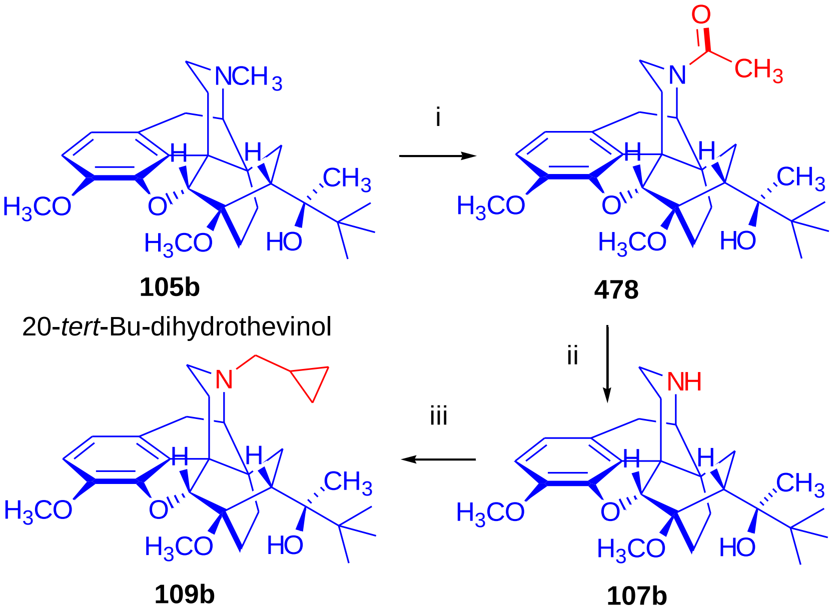

2.4. Figure S4 The Hudlický route for preparation of buprenorphine methylether

Reagents and conditions: (i): $Pd(OAc)_2$, $Cu(OAc)_2$, AcO, dioxane, 80 °C, air or O, 23 h, 95 %; (ii): Schwartz reagent: chlorodicycliopentadienylhydrozyrconium (Cp_2ZrHCl, 3 equiv.), THF, room temperature, 40 min, 90 %; (iii): cyclopropylmethyl bromide, $NaHCO_3$, *N*-methylpyrrolidine, 85 °C, 18 h, 88 %

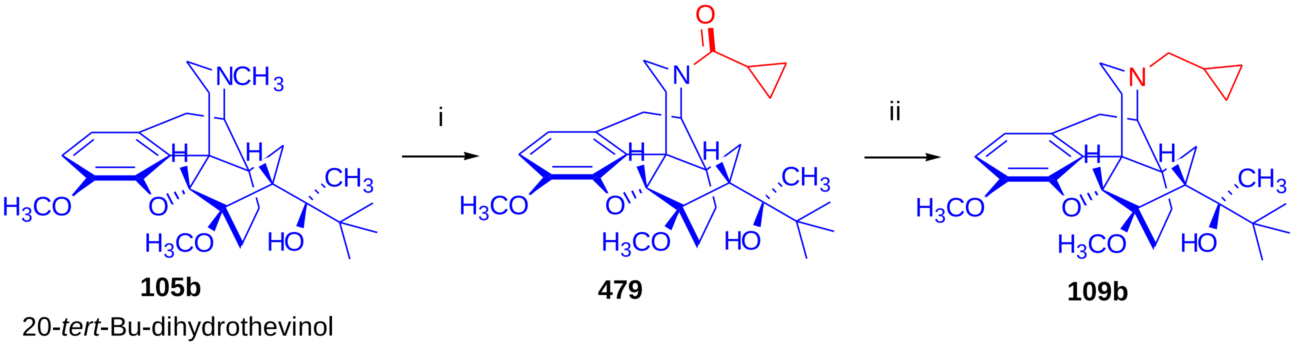

2.5. Figure S5 Synthesis of BPN methylether from 20-*tert*-butyl-dihydrothevinol using the *N*-demethylation/*N*-acylation reaction sequence

Reagents and conditions: (i): cyclopropanecarboxylic anhydride, $Pd(OAc)_2$, air or O_2, 100 °C, dioxane, 40-48 h, 90-95 %; (ii): LiAlH4, THF, 70 °C, 72 %, or Karstedt's catalyst, polymethylhydrosiloxane (PMHS), 87 %

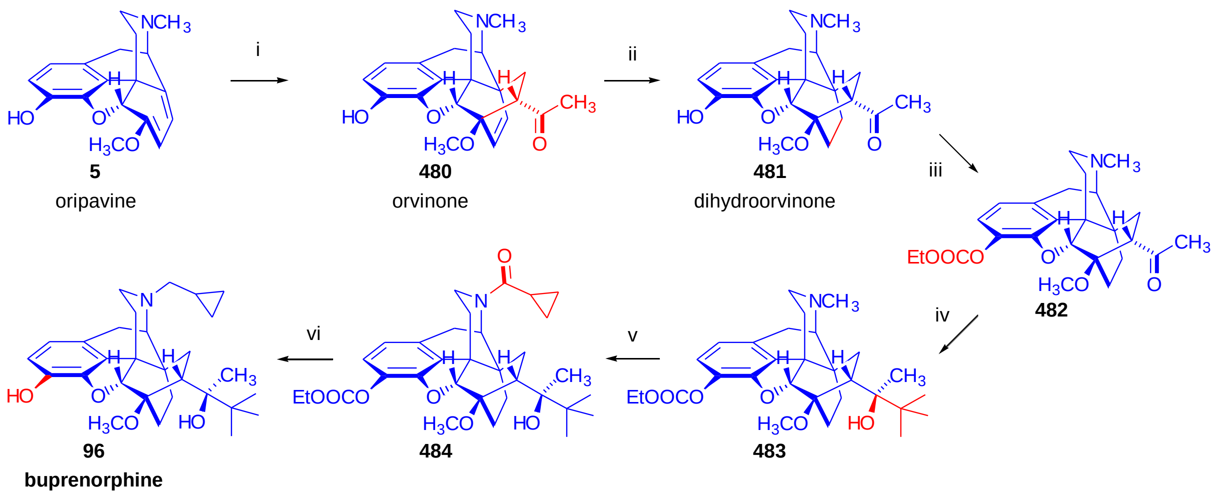

2.6. Figure S6 Synthesis of buprenorphine from oripavine

Reagents and conditions: (i): methyl vinyl ketone, toluene, 80 °C, 75%; (ii): H_2, 10% Pd-C, tartaric acid, water, atmospheric pressure, 16 h, 80 °C, 84%; (iii): EtCOOCl, Et_3N, toluene, 40 °C, 82%; (iv): tert-BuMgCl, cyclohexane, THF, toluene, room temperature, 71%; (v): cyclopropanecarboxylic anhydride, $Pd(OAc)_2$, $Cu(OAc)_2$, dioxane, O_2, 80 °C, 12 h, 80%; (vi): *bis*(2methoxyethoxy)aluminium hydride, THF, toluene, 80 °C, 30 min, 81%.

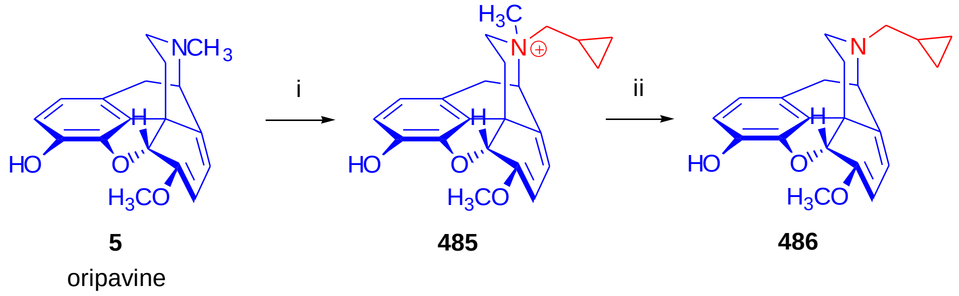

2.7. Figure S7 Synthesis of N^{17}-cyclopropylmethyl-nororipavine from oripavine

Reagents and conditions: (i): cyclopropylmethyl bromide, DMF, 80 °C, 94%; tert-dodecanethiol, EtONa, DMSO, 80 °C, 53%

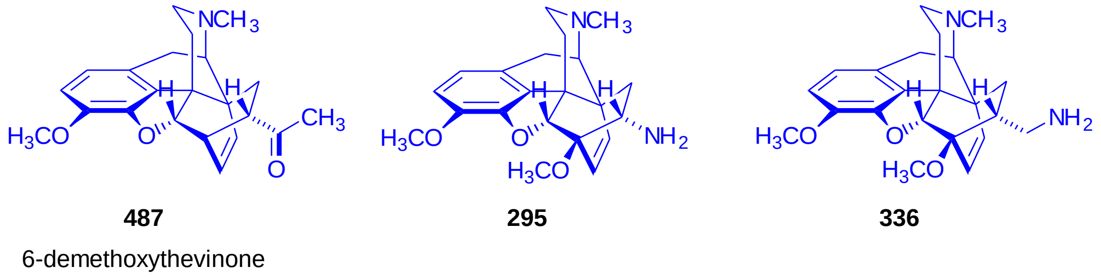

2.8. Figure S8 Chemical structures of non-acid-sensitive 6,14-ethenomorphinans 3-*O*-demethylated with boron tribromide
